# Effects of a Lifestyle Intervention in Routine Care on Prenatal Dietary Behavior—Findings from the Cluster-Randomized GeliS Trial

**DOI:** 10.3390/jcm8070960

**Published:** 2019-07-02

**Authors:** Julia Günther, Julia Hoffmann, Julia Kunath, Monika Spies, Dorothy Meyer, Lynne Stecher, Eva Rosenfeld, Luzia Kick, Kathrin Rauh, Hans Hauner

**Affiliations:** 1 Else Kröner-Fresenius-Centre for Nutritional Medicine, Klinikum rechts der Isar, Technical University of Munich, Georg-Brauchle-Ring 62, Munich 80992, Bavaria, Germany; 2 Competence Centre for Nutrition (KErn), Am Gereuth 4, Freising 85354, Bavaria, Germany

**Keywords:** lifestyle intervention, pregnancy, gestational weight gain (GWG), diet, exercise, dietary behavior, nutrition, obesity prevention

## Abstract

The antenatal lifestyle and excessive gestational weight gain (GWG) modify the risk of obstetric complications, maternal weight retention, and the risk of obesity for the next generation. The cluster-randomized controlled “Healthy living in pregnancy” (GeliS) study, recruiting 2286 women, was designed to examine whether a lifestyle intervention reduced the proportion of women with excessive GWG. Trained healthcare providers gave four counseling sessions covering a healthy diet, regular physical activity, and self-monitoring of GWG in the intervention group. In this secondary analysis, the effect on maternal dietary behavior was analyzed. Dietary behavior was assessed by means of a 58-item food frequency questionnaire in early and late pregnancy. The intervention resulted in a significant reduction in soft drink intake (*p* < 0.001) and an increase in the consumption of fish (*p* = 0.002) and vegetables (*p* = 0.023). With the exception of higher percentage energy from protein (*p* = 0.018), no effects of the intervention on energy and macronutrient intake were observed. There was no evidence for an overall effect on dietary quality measured with a healthy eating index. Some dietary variables were shown to be associated with GWG. In a routine prenatal care setting in Germany, lifestyle advice modified single aspects of dietary behavior, but not energy intake or overall dietary quality.

## 1. Introduction

Prepregnancy obesity as well as high gestational weight gain (GWG) can enhance the risk for pregnancy and obstetric complications [1,2,3] and are important determinants that elevate the long-term obesity risk in the offspring [4,5]. High GWG has additionally been reported to increase the risk for long-term maternal weight retention, and thus, to increase a mother’s obesity risk [6]. The Institute of Medicine (IOM) provided recommendations for adequate weight gain during pregnancy [7]. Nevertheless, the trend in prepregnancy obesity prevalence continues to rise concomitantly with the proportion of women with excessive GWG over the last decades [8,9].

While it is recommended to start obesity prevention preconceptually, there is still an urgent need to address adequate gestational weight gain. Over the past several years, lifestyle intervention approaches aimed at limiting GWG and reducing associated health complications have been initiated worldwide [10,11,12]. Although most intervention studies showed only modest effects, a recent individual participant data meta-analysis of 36 randomized trials reported a decrease in GWG by –0.7 kg [13]. The effect of lifestyle advice on behavioral outcomes is less known. Despite the fact that most trials include dietary advice, only a few of them reported dietary behavior comprehensively. For instance, the two largest published intervention studies observed no [14] or only modest effects on GWG [15]. However, study participants modified their diet in terms of energy, macronutrient, and fiber intake [15] or increased their consumption of fruit and vegetables and improved their overall dietary quality as measured by a healthy eating index (HEI) [16].

The large majority of lifestyle interventions in pregnancy took place in academic settings, and the number of studies integrating lifestyle advice using a settings-based approach is rather limited [17,18,19,20]. To address this, the GeliS (“Gesund leben in der Schwangerschaft”/healthy living in pregnancy) trial was initiated after a pilot study was successful in reducing the proportion of pregnant women with excessive GWG within the German primary care system [20]. By offering counseling on a healthy diet, regular physical activity, as well as monitoring of GWG, we aimed likewise to improve maternal lifestyle and to reduce excessive GWG. The primary findings of the trial have recently been published [21]. This secondary analysis evaluates the effectiveness of the GeliS lifestyle intervention on prenatal dietary behavior. We explored the mean daily consumption of food groups as well as energy and macronutrient intake. Dietary quality was assessed with an HEI. Additionally, potential associations between dietary behavior and GWG were explored.

## 2. Methods

### 2.1. Study Design

The GeliS study was designed as a prospective, multicenter, cluster-randomized, controlled, open intervention trial in five administrative regions of Bavaria, Germany. Five pairs of ten urban and rural areas were matched according to birth figures, sociodemographic, and geographic criteria, and cluster-randomized in one control and one intervention area per pair. The study was conducted in the German routine perinatal care setting within gynecological and midwifery practices.

The study procedures have been described previously [22] and were in accordance with the declaration of Helsinki as well as with local regulatory requirements and laws. The study protocol was approved by the ethics committee of the Technical University of Munich (project number 5653/13) and is registered at the ClinicalTrials.gov Protocol Registration System (NCT01958307).

### 2.2. Study Participants

Recruitment of pregnant study participants (≤12th week of gestation) was conducted by practice personnel at gynecological and midwifery practices between 2013 and 2015. If all inclusion criteria (singleton pregnancy, body mass index (BMI) between 18.5 and 40.0 kg/m^2^, aged between 18 and 43 years, sufficient German language skills) were fulfilled, women provided written informed consent for study participation. Those with severe illnesses and multiple or complicated pregnancies were excluded from study participation [22].

The control group (C) received routine prenatal care and a leaflet providing general information on a healthy lifestyle during pregnancy. In the intervention group (IV), routine care was complemented by a comprehensive lifestyle intervention program.

### 2.3. The Lifestyle Intervention Program

Pregnant women receiving the intervention program attended three individual face-to-face appointments during pregnancy (12th–16th, 16th–20th, and 30th–34th week of gestation) and one 68 weeks after delivery. After a specific training, counseling sessions were performed by midwives, gynecologists, and medical personnel in their practice rooms. The sessions, lasting 30–45 min each, were conducted alongside routine antenatal care visits. Recommendations on a healthy balanced diet, regular physical activity, and self-monitoring of GWG were provided. Dietary counseling included general recommendations on healthy eating principles according to the “Healthy Start-Young Family Network”, information on energy, macro- and critical micronutrient requirements during pregnancy, advice on the prevention of foodborne diseases, and personalized feedback on dietary behavior [23]. More information about the contents of the intervention program is provided in the published study protocol [22].

### 2.4. Study Outcomes

The primary aim of the GeliS study was to reduce the proportion of women with excessive GWG as defined by the IOM [7]. Results of the primary and selected secondary outcomes, including the incidence of gestational diabetes mellitus and other pregnancy and obstetric complications, have been published recently [21]. This secondary analysis focuses on the effectiveness of the lifestyle intervention in modifying dietary behavior of pregnant women and explores potential associations between dietary behavior and gestational weight gain.

### 2.5. Data Collection and Processing

Baseline characteristics were collected using a screening questionnaire at study entry. Body weight data were collected from maternity records. GWG was defined as the difference between maternal weight at the last prenatal visit and the first prenatal visit.

Dietary data were collected twice during pregnancy in both groups (baseline data ≤12th week of gestation, second assessment >29th week of gestation). A self-administered slightly modified version of the validated food frequency questionnaire (FFQ) developed for the “German Health Examination Survey for Adults” (DEGS) study by the Robert Koch Institute, Berlin, Germany, was applied [24]. Questions assessed dietary behavior patterns over the previous four weeks. The modified version consisted of 54 questions on consumption frequency and portion size of food items as well as four additional questions about specific food choices and dietary behaviors (e.g., vegetarianism, frequency of fresh food preparation). For each of the 54 food items, participants ranked consumption frequency on an 11-point scale ranging from “never” to “more than five times per day”. Portion sizes were given in usual measures including plates, bowls, cups, glasses, spoons, and pieces. Mean daily intake of food items was calculated according to the evaluation scheme provided by the developers of the DEGS-FFQ. Items were grouped to 17 food groups including nonalcoholic beverages, caffeinated beverages, soft drinks, alcoholic drinks, vegetables, fruit, cereal, side dishes, nuts, dairy products, cheese, eggs, fish, meat products, fats, sweets and snacks, and fast food. Questionnaires were excluded in the food group analysis if reported amounts of more than 20 of the 54 food items were missing. Questionnaire data of women reporting very high daily intakes (either liquids >15 kg, or solid foods >10 kg, or both liquids >4 kg and solid foods >6 kg) were considered implausible and thus excluded from analyses due to overreporting of food intake [25].

Energy, macronutrient, and fiber intake were estimated based on dietary information according to the German food composition database (“Bundeslebensmittelschlüssel”, version 3.02) using OptiDiet PLUS software (version 6.0, GOE mbH, Linden, Germany). Some questions in the FFQ comprised multiple food items. In these cases, data of typical distribution patterns in the consumption of these food items from the German National Consumption Survey II (NVS II) were taken into account for the estimation of energy and macronutrient intake [26]. If estimated daily energy intake was <4500 kJ or >20,000 kJ, women were excluded from energy and macronutrient analyses due to under- or overreporting of energy intake [27].

To assess the quality of the diet, a healthy eating index was calculated based on the food groups derived from the administered FFQ. The DEGS-HEI was developed at the Robert Koch Institute based on the DEGS-FFQ [28] and rates the intake of 14 food groups according to the adherence to the German Nutrition Society (DGE) recommendations on a healthy diet. Each food group is scored with 0 (no adherence to recommendations) to 100 (very high adherence to recommendations) points, and a combined HEI score was calculated from the mean group scores (0 to 100 points).

### 2.6. Statistical Analysis

Power calculation was based on the primary endpoint and has been described previously [22]. All women providing at least one valid questionnaire were included in the dietary analyses, with the exception of those that had a miscarriage, pregnancy termination, severe pregnancy complications, or in case of maternal death. Analyses relating to GWG further excluded participants with preterm delivery (<37th week of gestation).

For the comparison of dietary behavior between groups, linear and logistic regression models were fit with generalized estimating equations (GEEs) [29]. Models were adjusted for prepregnancy BMI category, age, parity, and baseline food intake of respective items. Exploratory subgroup analyses were performed according to the women’s age, prepregnancy BMI category, and educational level with similar models. An interaction of group with these factors was considered in order to screen for factors influencing the treatment effect. Group differences are presented as estimated mean differences or odds ratios with 95% confidence interval (CI). For the analysis of changes in dietary behavior from baseline to late pregnancy, linear mixed models for repeated measures were applied. Models were adjusted for prepregnancy BMI category, parity, and age of the women. Associations of dietary data with gestational weight gain were analyzed with linear regression models. For this exploration, groups were pooled to form one cohort, and models were additionally adjusted for group assignment. All analyses were performed using SPSS software (IBM SPSS Statistics for Windows, version 24.0, IBM Corp, Armonk, NY, USA).

## 3. Results

### 3.1. Study Participants and Baseline Characteristics

**Figure 1:**
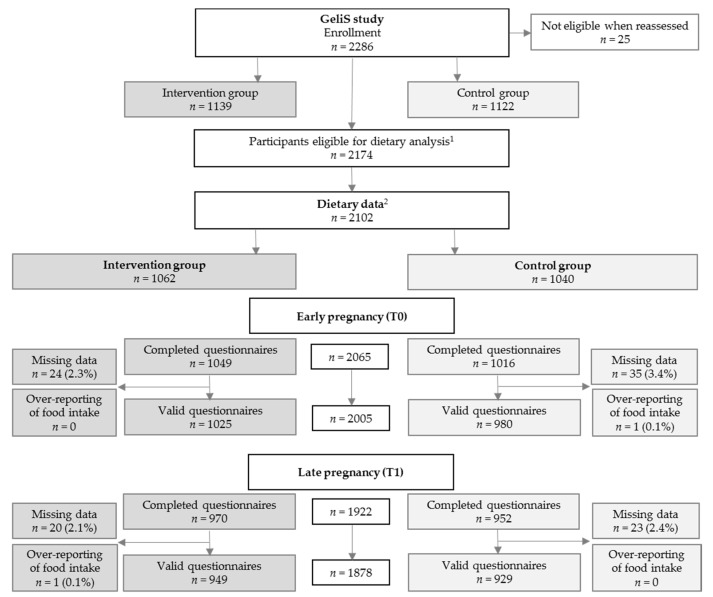
Participant flow for dietary analysis within the GeliS trial. GeliS, “Gesund leben in der Schwangerschaft”/healthy living in pregnancy; T0: assessment before the 12th week of gestation; T1: assessment after the 29th week of gestation. ^1^ Women without miscarriages, late loss of pregnancy, terminations, pregnancy complications that interfere with the intervention, maternal deaths; ^2^ Women providing dietary data at T0 or T1.

Initially, 2286 women were recruited for participation in the GeliS trial. After exclusion of 25 women, who were determined to be ineligible after reassessment of in- and exclusion criteria, 1139 women received lifestyle counseling and 1122 received routine prenatal care (Figure 1). After excluding women with a miscarriage, termination of pregnancy, and severe complications, 2174 women were eligible for dietary analysis. Dietary data were available for a total of 2065 (95.0%) women at baseline (T0) and 1922 (88.4%) in late pregnancy (T1). A total of 2005 (92.2%, T0) and 1878 (86.4%, T1) valid FFQs were considered for the analysis of food consumption data (Figure 1). For the analysis of energy and macronutrient intake, further questionnaires were excluded due to under- and overreporting of the estimated daily energy intake in early (*n* = 185) and late pregnancy (*n* = 124), resulting in a sample of 1820 (83.7%, T0) and 1754 (80.7%, T1) women for this specific analysis.

Baseline characteristics of study participants providing any dietary data (*n* = 2102) are shown in Table 1. Groups were largely comparable concerning mean prepregnancy age (30.1 vs. 30.3 years), self-reported body weight (68.4 kg vs. 67.9 kg), and BMI (24.4 kg/m^2^ vs. 24.3 kg/m^2^). The total sample consisted of 65.3% women with normal weight, 22.7% with overweight, and 12.0% with obesity. In both groups, educational level was comparable, and the majority of women were born in Germany. In the IV, the proportion of nulliparous women was higher compared to the control (62.2% vs. 53.6%).

### 3.2. Food Intake

Mean daily reported intake of 17 food groups in IV and C is shown in Table 2. In late pregnancy, the lifestyle intervention group showed a higher consumption of vegetables and fish compared to women in the control group (adjusted effect size for vegetable intake 19.83 g/day, 95% CI 2.75 to 36.91 g/day, *p* = 0.023; adjusted effect size for fish intake 1.82 g/day, 95% CI 0.68 to 2.96 g/day, *p* = 0.002). While there was no evidence that the intervention influenced total intake of major food groups such as beverages, dairy products or cereal, the soft drink intake of women of the IV was significantly reduced at T1 compared to the C (155.45 vs. 235.36 mL/day in late pregnancy, *p* < 0.001). In late pregnancy, intake of caffeinated beverages was by trend lower in women allocated to the lifestyle intervention group compared to women in the C (adjusted effect size –6.38 mL/day, 95% CI –12.85 to 0.10 mL/day, *p* = 0.054). Total beverage as well as fast food consumption decreased over the course of pregnancy in both groups. Reported intake of dairy products and sweets and snacks increased over time in both IV and C (Table 2).

A summary of specific dietary behaviors recorded with the FFQ is given in Table 3. Among women receiving lifestyle counseling, a higher proportion showed any intake of whole-grain bread (*p* = 0.002) and chose low-fat varieties of milk or yogurt *(p* < 0.001) as well as cheese or sausage (*p* < 0.001) compared to the control. Additionally, more women in the IV were vegetarian (*p* = 0.008), and they were more likely to choose olive or rapeseed oil over other oils and fats (*p* < 0.001). In both groups, the number of participants preparing fresh food on an almost daily basis increased during the course of pregnancy (*p* < 0.001 for both groups).

### 3.3. Energy and Macronutrient Intake

There was no evidence of major differences in energy and macronutrient intake between groups (Table 4). Mean energy intake was 1974 kcal/day and 1945 kcal/day in IV and C, respectively, at T0 and 2000 kcal/day and 2011 kcal/day at T1. Percentage energy (E%) from protein was slightly higher in women receiving lifestyle advice (adjusted effect size 0.44 E%, 95% CI 0.08 to 0.81 E%, *p* = 0.018) compared to the C. In both groups, E% from carbohydrates decreased over time, whereas E% from fats increased (Table 4). Alcohol intake was low in both groups and decreased over the course of pregnancy. Fiber intake was comparable between the two groups (Table 4).

### 3.4. Healthy Eating Index

No group difference in overall dietary quality rated by means of the healthy eating index was observed (Table 4). Dietary quality increased over time in both groups (*p* < 0.001). Details about group scores of the single food categories are given in Supplementary Table S1. Subgroup analyses according to women’s prepregnancy age, BMI category, and educational level showed evidence of a significant difference between IV and C in the subgroup of young women aged 18–25 years (adjusted effect size 3.39, 95% CI 1.36 to 5.43, *p* = 0.001) and women with general secondary education (adjusted effect size 2.01, 95% CI 1.29 to 2.74, *p* < 0.001, Table 5). There was evidence for an influence of prepregnancy age (interaction *p* = 0.001) as well as BMI category (interaction *p* = 0.011) on the intervention effect. Irrespective of group allocation, the HEI was statistically significantly influenced by age, BMI, and educational level in early as well as late pregnancy (*p* < 0.001 respectively, data not shown).

### 3.5. Dietary Behavior and Gestational Weight Gain

Several aspects of dietary behavior were positively associated with total GWG. In Table 6, effect sizes of associations between GWG and typical portion sizes of food groups are shown. Evidence of significant positive associations was found for intake of cheese (*p* = 0.045) and eggs (*p* = 0.013) in early pregnancy, and for dairy products (*p* < 0.001), processed meat (*p* = 0.028), and sweets and snacks (*p* = 0.001) in late pregnancy. Fast food consumption promoted weight gain at both early (*p* < 0.001) and late pregnancy (*p* = 0.007). Choosing low-fat milk and yogurt was associated with increased GWG at both T0 (*p* = 0.005) and T1 (*p* < 0.001), whereas the selection of low-fat cheese and sausage was only associated with weight gain during late pregnancy (*p* = 0.009). There was no evidence of an association of either early energy intake or macronutrient composition with GWG, whereas late energy intake (*p* < 0.001) as well as sugar consumption (*p* = 0.004) was shown to be positively associated with GWG.

## 4. Discussion

To the best of our knowledge, the GeliS trial is the largest intervention study worldwide evaluating the effect of dietary and physical activity counseling within the setting of routine prenatal care. Although lifestyle counseling was not successful in limiting GWG, the findings described herein suggest effects on several aspects of the maternal diet.

However, there was no evidence of a modification in energy intake, which is in line with the missing effect of the intervention on GWG [21]. Effects of previously conducted lifestyle interventions on caloric intake are heterogeneous [15,16,30,31,32,33]. The LIMIT trial, which included 2212 pregnant women with overweight or obesity, provided counseling on a healthy lifestyle but also could not demonstrate an effect on energy intake [16]. By contrast, the UPBEAT trial, with a cohort of 1555 women with obesity, was able to show a reduction in daily energy consumption in the intervention group [15].

As demonstrated in both the LIMIT and UPBEAT trial, as well as in the GeliS study, specific dietary components and behaviors seem to be more easily modifiable than overall energy intake. Some of the messages communicated as part of the GeliS lifestyle counseling were followed by women in the IV, including, for instance, a reduction in the consumption of sugar-sweetened beverages. Mean daily soft drink consumption in the IV was reduced to 155 mL in late pregnancy, complying with the recommendation not to exceed one glass per day, whereas mean consumption in the control group was higher (235 mL). Consumption of sugar-sweetened beverages has been suggested to increase the risk for complications, such as gestational diabetes [34], and to reduce birth weight [35] but to increase early childhood BMI [36]. Furthermore, the intervention successfully increased maternal mean vegetable and fish intake. This was similarly observed in other lifestyle intervention trials for vegetable [16,30,31] and fish consumption [37]. Additionally, lifestyle counseling in the GeliS trial effectively promoted the choice of rapeseed and olive oil over other oils and fats. Home-cooking was not shown to be influenced by group assignment, and the frequency of fresh cooking was high in both groups. Further effects included a higher rate of women choosing whole-grain bread and low-fat alternatives, which is in line with the observations of others [31].

Nevertheless, the reported changes did not lead to a significant improvement in overall dietary quality, as measured with an HEI. Dietary quality increased over time in both groups, but no consistent intervention effect could be identified. The success of the intervention in modifying dietary quality was, however, significantly influenced by prepregnancy BMI and maternal age. Those who particularly seemed to benefit from lifestyle counseling were young women, women with general secondary education and by trend normal-weight women. Dietary counseling content in future studies may need to be adapted for certain subgroups of women to provide better support and guidance in order to optimize its success. Irrespective of group assignment, dietary quality appeared to be dependent on BMI and sociodemographic factors such as age and educational level, consistent with previously reported literature [38]. Independent of subgroups, other lifestyle interventions showed moderate beneficial changes in dietary quality indices [16,39]. Nevertheless, these and most other intervention studies aiming to improve maternal lifestyle have been conducted in academic surroundings. Achieving effective behavioral changes under “real-life” conditions remains a challenge but is essential to be applicable at the population level.

The GeliS study was conducted in the German routine prenatal care setting and represents a true public health approach. Counseling sessions as well as collection of dietary data were performed within gynecological and midwifery practices. The fact that moderate changes in dietary behavior were achievable under realistic conditions supports the GeliS setting approach for health promotion, albeit no effect on GWG was observed. The preceding pilot study FeLIPO (“Feasibility of a Lifestyle Intervention in Pregnancy to Optimize maternal weight development”) showed that a lifestyle intervention in a routine care setting can successfully normalize energy intake and simultaneously beneficially influence GWG [20]. This finding could not be repeated in the GeliS study. However, counseling in the FeLIPO trial was performed by a dietary expert, while trained practice personnel provided counseling in the GeliS study. Involving dietary experts could strengthen the concept and possibly induce changes in dietary behavior that are sufficient to reduce excessive GWG.

In our cohort, increased intake of several dietary components was related to higher total GWG, including mainly animal products such as cheese and other dairy products, eggs and processed meat, but also sweets, snacks, and fast food. Some observational studies have similarly linked specific food groups, such as dairy products, or energy-dense food groups such as sweets and fast food or fried food to high or excessive GWG [40,41]. Nonetheless, evidence thus far is still sparse and inconclusive. Generally, the most significant dietary factor influencing pregnancy weight gain seems to be maternal energy intake [42], although even for this factor, the data are not completely consistent [43]. Energy as well as sugar intake were significantly associated with increased GWG in the GeliS cohort in late but not in early pregnancy. An effect of sugar consumption on GWG has also been suggested in other trials [44,45]. Interestingly, choosing low-fat alternatives seemed to promote weight gain in the GeliS study sample. This could potentially be related to a higher total food intake in the subgroup of women who usually chose low-fat products, although associations remained statistically significant after post-hoc adjustment for energy intake. Choosing low-fat products is often part of dietary recommendations and was also encouraged during our counseling sessions. Concluding from the presented findings, lifestyle counseling approaches may need to reconsider whether they should continue to encourage pregnant women to consume low-fat products.

In addition to its uniqueness as the first large-scaled public health intervention trial in this field, the GeliS study reports several other particular strengths. The dietary behavior of women across multiple BMI categories was assessed, largely representative of all German pregnant women. Baseline characteristics were comparable between groups, except for parity, which was adjusted for in all analyses. Dietary behavior was comprehensively assessed, including reporting the daily consumption of food groups, estimating energy and macronutrient intake and calculating an HEI specifically developed to interpret the FFQ used in this study [28]. The presented findings help to evaluate the effects of dietary counseling during pregnancy and to provide insights into which components of prenatal intervention need to be emphasized in the future.

Nevertheless, the dietary assessment performed in the GeliS trial has some limitations. Compared to other dietary assessment methods, FFQs provide data on self-reported consumption rates and strongly depend on the participant’s memory. However, the DEGS-FFQ has previously been validated and has been applied to compare dietary intake between groups [24], as was done in this analysis. In the GeliS trial, conducted in a public health setting and with a large sample size, using an FFQ was a more realistic and feasible tool than applying more detailed methods. Moreover, requirements for study participants were low compared to using dietary records. Calculation of energy and macronutrient intake, however, provide only rough estimates of the actual intake, as the underlying FFQ has not been designed for this kind of analysis and contains some questions about food groups rather than single food items. In order to enhance the accuracy of this estimation, data of typical German distribution patterns in the consumption of relevant food items were taken into account. A principal limitation in this kind of trial is that participants in the control regions also received general recommendations on a healthy prenatal diet. As a result, women in C may have altered their dietary behavior to conform to general recommendations. Moreover, bias may have been introduced due to an increased awareness from filling in the questionnaires, which could potentially lead to under- or overreporting.

## 5. Conclusions

In the German routine prenatal care setting, lifestyle counseling was effective in inducing some beneficial dietary changes. The magnitude of these changes was, however, insufficient to improve overall dietary quality or to prevent excessive GWG. A stronger emphasis on energy intake with the addition of counseling provided by dietary experts, e.g., dietitians, could help to achieve more pronounced effects. Our results suggested positive associations between a number of food groups, such as animal products, sweets, snacks and fast food, and pregnancy weight gain. These findings may help to give more specific dietary advice to pregnant women. A planned 5-year follow-up of the GeliS mother–child cohort will provide the opportunity to evaluate whether the observed changes in prenatal dietary behavior will have long-term effects on offspring and maternal health outcomes.

## Figures and Tables

**Table 1:** Baseline characteristics of study participants with available dietary data (mean ± SD or proportions).

	Intervention (*n* = 1062)	Control (*n* = 1040)	Total (*n* = 2102)
Prepregnancy age (years)	30.1 ± 4.3	30.3 ± 4.6	30.2 ± 4.5
Prepregnancy weight (kg)	68.4 ± 13.1	67.9 ± 13.7	68.2 ± 13.4
Prepregnancy BMI (kg/m^2^)	24.4 ± 4.4	24.3 ± 4.6	24.4 ± 4.5
Prepregnancy BMI category (*n* (%))			
BMI 18.5–24.9 kg/m^2^	685/1062 (64.5%)	687/1040 (66.1%)	1372/2102 (65.3%)
BMI 25.0–29.9 kg/m^2^	253/1062 (23.8%)	225/1040 (21.6%)	478/2102 (22.7%)
BMI 30.0–40.0 kg/m^2^	124/1062 (11.7%)	128/1040 (12.3%)	252/2102 (12.0%)
Educational level			
General secondary school	157/1061 (14.8%)	173/1036 (16.7%)	330/2097 (15.7%)
Intermediate secondary school	454/1061 (42.8%)	430/1036 (41.5%)	884/2097 (42.2%)
High school	450/1061 (42.4%)	433/2096 (41.8%)	883/2097 (42.1%)
Country of birth (*n* (%))			
Germany	933/1061 (87.9%)	930/1037 (89.7%)	1863/2098 (88.8%)
Others	128/1061 (12.1%)	107/1037 (10.3%)	235/2098 (11.2%)
Nulliparous (*n* (%))	661/1062 (62.2%)	557/1039 (53.6%)	1218/2101 (58.0%)
Living with a partner (*n* (%))	1023/1058 (96.7%)	988/1037 (95.3%)	2011/2095 (96.0%)
Full-time employed	568/1048 (54.2%)	514/1031 (49.9%)	1082/2079 (52.0%)

BMI: body mass index.

**Table 2:** Mean daily food intake in the intervention and control groups.

	Time Point	Intervention Group		Control Group		Adjusted Effect Size ^1^ (95% CI)	Adjusted *p* Value ^1^
		*n*	Mean ± SD	*n*	Mean ± SD		
Beverage consumption (mL/day)	T0	1025	3414.37 ± 2176.67	979	3434.05 ± 2176.93		
	T1	949	3029.63 ± 1883.61	928	3060.04 ± 1965.45	–34.46(–226.08,157.15)	0.724
	Time effect	*p* < 0.001 ^2^		*p* < 0.001 ^2^			
Caffeinated beverages (mL/day)	T0	1024	118.02 ± 172.24	979	142.91 ± 204.40		
	T1	949	134.59 ± 152.65	927	148.00 ± 177.60	–6.38(–12.85,0.10)	0.054
	Time effect	*p* = 0.005 ^2^		*p* = 0.283 ^2^			
Soft drinks (mL/day)	T0	1025	207.74 ± 600.99	979	247.50 ± 676.18		
	T1	949	155.45 ± 439.32	927	235.36 ± 641.33	–57.03(–86.24,–27.83)	<0.001
	Time effect	*p* = 0.004 ^2^		*p* = 0.276 ^2^			
Alcoholic drinks (mL/day)	T0	1023	11.13 ±40.88	974	14.01 ± 49.85		
	T1	947	13.80 ± 39.28	922	15.62 ± 46.99	–1.31(–5.36,2.75)	0.527
	Time effect	*p* = 0.055 ^2^		*p* = 0.416 ^2^			
Vegetables (g/day)	T0	1022	178.63 ± 146.58	980	171.73 ± 151.76		
	T1	949	201.04 ± 159.94	927	175.72 ± 160.22	19.83(2.75,36.91)	0.023
	Time effect	*p* < 0.001 ^2^		*p* = 0.295 ^2^			
Fruit (g/day)	T0	1022	328.57 ± 324.63	980	296.36 ± 304.94		
	T1	948	315.50 ± 298.02	928	291.61 ± 271.70	4.73(–6.33,15.79)	0.402
	Time effect	*p* =0.243 ^2^		*p* = 0.761 ^2^			
Nuts (g/day)	T0	1021	2.54 ± 5.78	975	1.86 ± 4.05		
	T1	946	2.98 ± 6.00	925	2.60 ± 5.46	0.08(–0.19,0.35)	0.563
	Time effect	*p* =0.018 ^2^		*p* < 0.001 ^2^			
Cereal (g/day)	T0	1025	121.76 ± 83.11	979	116.06 ± 80.79		
	T1	949	115.01 ± 69.49	929	112.87 ± 70.66	–0.81(–7.75,6.13)	0.818
	Time effect	*p* =0.014 ^2^		*p* = 0.291 ^2^			
Side dishes (g/day)	T0	1024	142.98 ± 80.74	980	144.57 ± 96.03		
	T1	949	141.50 ± 78.85	929	139.47 ± 94.80	1.83(–3.78,7.44)	0.523
	Time effect	*p* =0.378 ^2^		*p* = 0.128 ^2^			
Dairy products (g/day)	T0	1025	327.66 ± 277.66	979	333.17 ± 384.98		
	T1	949	397.52 ± 335.81	929	372.16 ± 365.10	24.56(–5.13,54.26)	0.105
	Time effect	*p* < 0.001 ^2^		*p* = 0.002 ^2^			
Cheese (g/day)	T0	1021	26.58 ± 34.55	975	26.77 ± 36.41		
	T1	944	25.76 ± 28.49	923	25.42 ± 29.30	–0.68(–4.24,2.89)	0.709
	Time effect	*p* = 0.566 ^2^		*p* = 0.983 ^2^			
Eggs (g/day)	T0	1019	14.20 ± 15.60	973	15.03 ± 17.99		
	T1	945	14.20 ± 21.86	926	13.85 ± 15.67	0.28(–1.60, 2.15)	0.774
	Time effect	*p* = 0.966 ^2^		*p* = 0.096 ^2^			
Fat spread (g/day)	T0	1016	5.04 ± 6.57	977	5.14 ± 6.21		
	T1	946	5.39 ± 6.05	922	5.50 ± 5.77	–0.15(–0.67,0.37)	0.561
	Time effect	*p* = 0.129 ^2^		*p* = 0.032 ^2^			
Fish (g/day)	T0	1025	12.78 ± 16.10	979	12.74 ± 13.86		
	T1	947	14.51 ± 12.13	926	12.49 ± 12.52	1.82(0.68, 2.96)	0.002
	Time effect	*p* = 0.001 ^2^		*p* = 0.729 ^2^			
Meat and meat products (g/day)	T0	1024	82.14 ± 57.35	980	80.31 ± 56.53		
	T1	949	85.49 ± 51.48	929	84.79 ± 52.00	–0.93(–5.46, 3.61)	0.689
	Time effect	*p* = 0.113 ^2^		*p* = 0.010 ^2^			
Sweets and snacks (g/day)	T0	1025	70.14 ± 55.15	980	71.40 ± 68.25		
	T1	949	88.76 ± 70.00	929	89.85 ± 65.62	–0.99(–8.19, 6.21)	0.788
	Time effect	*p* < 0.001 ^2^		*p* < 0.001 ^2^			
Fast food (g/day)	T0	1024	46.80 ± 37.12	980	48.13 ± 35.77		
	T1	949	43.14 ± 31.22	929	44.52 ± 30.46	–1.56(–3.84,0.72)	0.180
	Time effect	*p* = 0.001 ^2^		*p* = 0.002 ^2^			

BMI: body mass index; T0: baseline assessment before the 12th week of gestation; T1: assessment after the 29th week of gestation. ^1^ Linear regression models fit using generalized estimating equations adjusted for prepregnancy BMI, age, parity, and baseline intake (T0); ^2^ Linear mixed models for repeated measures adjusted for prepregnancy BMI, age, and parity.

**Table 3:** Specific dietary choices in the intervention and control groups.

	Time Point	Intervention Group		Control Group		Adjusted Effect Size ^1^ (95% CI)	Adjusted *p* Value ^1^
		*n*	%	*n*	%		
Whole grain bread	T0	953/1019	93.5%	911/975	93.4%		
	T1	918/943	97.3%	866/922	93.9%	2.95(1.49,5.87)	0.002
	Time effect	*p* < 0.001 ^2^		*p* = 0.740 ^2^			
Low-fat milk/yoghurt	T0	725/1006	72.1%	675/965	69.9%		
	T1	692/943	73.4%	638/915	69.7%	1.28(1.12,1.47)	<0.001
	Time effect	*p* = 0.369 ^2^		*p* = 0.662 ^2^			
Low-fat cheese/sausage	T0	531/976	54.4%	496/939	52.8%		
	T1	517/923	56.0%	465/902	51.6%	1.27(1.20,1.35)	<0.001
	Time effect	*p* = 0.289 ^2^		*p* = 0.199 ^2^			
Sugar in coffee/tea	T0	382/993	38.5%	390/950	41.1%		
	T1	335/911	36.8%	359/891	40.3%	0.86(0.72,1.03)	0.098
	Time effect	*p* = 0.119 ^2^		*p* = 0.811 ^2^			
Rapeseed oil and olive oil (for meat and fish)	T0	474/888	53.4%	477/833	57.3%		
	T1	478/786	60.8%	447/796	56.2%	1.72(1.57,1.89)	<0.001
	Time effect	*p* < 0.001 ^2^		*p* = 0.553 ^2^			
Rapeseed oil and olive oil (for vegetables)	T0	507/860	59.0%	497/840	59.2%		
	T1	514/780	65.9%	475/793	59.9%	1.55(1.32,1.82)	<0.001
	Time effect	*p* < 0.001 ^2^		*p* = 0.825 ^2^			
Cooking at least 5 times per week	T0	586/1022	57.3%	545/975	55.9%		
	T1	604/940	64.3%	575/924	62.2%	1.20(1.00,1.43)	0.050
	Time effect	*p* < 0.001 ^2^		*p* < 0.001 ^2^			
Vegetarian	T0	67/1019	6.6%	68/969	7.0%		
	T1	57/938	6.1%	49/917	5.3%	1.45(1.10,1.92)	0.008
	Time effect	*p* = 0.321 ^2^		*p* = 0.029 ^2^			

BMI: body mass index; T0: baseline assessment before the 12th week of gestation; T1: assessment after the 29th week of gestation. ^1^ Logistic regression models fit using generalized estimating equations adjusted for prepregnancy BMI, age, parity, and baseline intake (T0); ^2^ Linear mixed models for repeated measures adjusted for prepregnancy BMI, age, and parity.

**Table 4:** Mean daily intake of energy and macronutrients in the intervention and control groups.

	Time Point	Intervention Group		Control Group		Adjusted Effect Size ^1^ (95% CI)	Adjusted *p* Value ^1^
		*n*	Mean ± SD	*n*	Mean ± SD		
Energy (kcal/day)	T0	940	1974.22 ± 618.56	880	1944.66 ± 661.60		
	T1	892	2000.39 ± 606.90	862	2010.79 ± 646.59	–17.67(–115.71,80.36)	0.724
	Time effect	*p* = 0.137 ^2^		*p* < 0.001 ^2^			
Carbohydrates (E%)	T0	940	57.13 ± 8.07	880	57.18 ± 8.13		
	T1	892	54.66 ± 7.01	862	55.46 ± 7.41	–0.31(–1.50,0.88)	0.606
	Time effect	*p* < 0.001 ^2^		*p* < 0.001 ^2^			
Saccharose (g/day)	T0	940	54.84 ± 30.72	880	54.48 ± 33.47		
	T1	892	56.08 ± 27.93	862	57.82 ± 31.18	–0.78(–4.79,3.23)	0.702
	Time effect	*p* = 0.206 ^2^		*p* = 0.001 ^2^			
Protein (E%)	T0	940	16.14 ± 3.11	880	16.16 ± 3.38		
	T1	892	16.55 ± 3.03	862	15.95 ± 2.99	0.44(0.08,0.81)	0.018
	Time effect	*p* < 0.001 ^2^		*p* = 0.171 ^2^			
Fat (E%)	T0	940	26.65 ± 6.41	880	26.49 ± 6.37		
	T1	892	28.77 ± 5.78	862	28.55 ± 6.00	–0.07(–1.01,0.87)	0.888
	Time effect	*p* < 0.001 ^2^		*p* < 0.001 ^2^			
Saturated fat (g/day)	T0	940	26.55 ± 11.66	880	26.01 ± 12.07		
	T1	892	29.72 ± 12.92	862	29.58 ± 12.96	–0.45(–2.38,1.48)	0.647
	Time effect	*p* < 0.001 ^2^		*p* < 0.001 ^2^			
Saturated fat (E%)	T0	940	12.61 ± 3.46	880	12.55 ± 3.44		
	T1	892	13.77 ± 3.17	862	13.69 ± 3.31	–0.05(–0.54,0.44)	0.845
	Time effect	*p* < 0.001 ^2^		*p* < 0.001 ^2^			
MUFA (g/day)	T0	940	17.44 ± 7.48	880	16.95 ± 7.60		
	T1	892	19.19 ± 8.11	862	19.22 ± 8.16	–0.43(–1.52,0.66)	0.441
	Time effect	*p* < 0.001 ^2^		*p* < 0.001 ^2^			
PUFA (g/day)	T0	940	6.24 ± 2.43	880	6.08 ± 2.43		
	T1	892	6.40 ± 2.38	862	6.35 ± 2.44	–0.08(–0.33,0.17)	0.517
	Time effect	*p* = 0.039 ^2^		*p* < 0.001 ^2^			
Cholesterol (mg/day)	T0	940	227.32 ± 97.71	880	226.11 ± 108.11		
	T1	892	241.21 ± 125.34	862	237.59 ± 106.56	2.19(–10.74,15.13)	0.740
	Time effect	*p* < 0.001 ^2^		*p* < 0.001 ^2^			
Fiber (g/day)	T0	940	24.82 ± 10.60	880	23.36 ± 10.54		
	T1	892	24.72 ± 10.10	862	23.25 ± 9.86	0.72(–0.46,1.90)	0.232
	Time effect	*p* = 0.814 ^2^		*p* = 0.996 ^2^			
Alcohol (g/day)	T0	940	0.20 ± 0.91	880	0.44 ± 2.32		
	T1	892	0.07 ± 0.24	862	0.11 ± 0.43	–0.03(–0.06,0.00)	0.068
	Time effect	*p* < 0.001 ^2^		*p* < 0.001 ^2^			
Healthy Eating Index	T0	1025	58.81 ± 8.60	980	57.54 ± 8.93		
	T1	949	59.33 ± 8.21	929	57.60 ± 8.52	1.05(–0.42,2.53)	0.162
	Time effect	*p* < 0.001 ^2^		*p* < 0.001 ^2^			

BMI: body mass index; MUFA: monounsaturated fatty acids; PUFA: polyunsaturated fatty acids; T0: baseline assessment before the 12th week of gestation; T1: assessment after the 29th week of gestation. ^1^ Linear regression models fit using generalized estimating equations adjusted for prepregnancy BMI, age, parity, and baseline intake (T0) ^2^ Linear mixed models for repeated measures adjusted for prepregnancy BMI, age, and parity.

**Table 5:** Healthy eating index in subgroups of the intervention and control groups after the 29th week of gestation (T1).

	Intervention Group		Control Group		Adjusted Effect Size ^1^ (95% CI)	Adjusted *p* Value ^1^	Interaction *p* Value
	*n*	Mean ± SD	*n*	Mean ± SD			
**Prepregnancy age category**							0.001 ^2^
Age 18–25 years	121	58.18 ± 7.84	131	53.78 ± 7.76	3.39 (1.36,5.43)	0.001	
Age 26–35 years	723	59.31 ± 8.13	669	57.84 ± 8.37	0.96 (–0.60,2.53)	0.228	
Age 36–43 years	105	60.80 ± 8.99	127	60.31 ± 8.82	–0.08 (–2.00,1.84)	0.934	
**Prepregnancy BMI category**							0.011 ^3^
BMI 18.5–24.9 kg/m^2^	612	60.06 ± 8.15	612	58.15 ± 8.46	1.45 (–0.12,3.03)	0.069	
BMI 25.0–29.9 kg/m^2^	228	58.70 ± 8.22	197	56.85 ± 8.90	0.89 (–1.20,2.98)	0.403	
BMI 30.0–40.0 kg/m^2^	109	56.55 ± 7.90	120	56.03 ± 7.95	–0.14 (–1.76,1.48)	0.867	
**Educational level**							0.644 ^4^
General secondary school	137	55.65 ± 8.77	147	54.54 ± 7.91	2.01 (1.29,2.74)	<0.001	
Intermediate secondary school	410	58.18 ± 7.71	396	56.75 ± 8.33	0.89 (–0.55,2.33)	0.226	
High school	401	61.75 ± 7.81	384	60.03 ± 8.21	1.31 (–0.18,2.81)	0.086	

BMI: body mass index; T0: baseline assessment before the 12th week of gestation; T1: assessment after the 29th week of gestation. ^1^ Linear regression models fit using generalized estimating equations adjusted for prepregnancy BMI, age, parity, and baseline assessment (T0); ^2^ Linear regression model fit using generalized estimating equations including group, age category and group x age category, adjusted for prepregnancy BMI, parity, and baseline assessment (T0); ^3^ Linear regression model fit using generalized estimating equations including group, BMI category and group x BMI category, adjusted for age, parity, and baseline assessment (T0); ^4^ Linear regression model fit using generalized estimating equations including group, educational level and group x educational level, adjusted for prepregnancy BMI age, parity, and baseline assessment (T0).

**Table 6:** Association between dietary components in early (T0) and late (T1) pregnancy with total gestational weight gain (kg).

	T0			T1		
	*n*	Adjusted Effect Size ^1^ (95% CI)	Adjusted *p* Value ^1^	*n*	Adjusted Effect Size ^1^ (95% CI)	Adjusted *p* Value ^1^
Soft drinks (200 mL/day)	1774	0.06 (–0.01,0.14)	0.097	1744	0.04 (–0.05,0.13)	0.347
Vegetables (150 g/day)	1772	0.16 (–0.08,0.39)	0.185	1744	0.05 (–0.17,0.27)	0.671
Fruit (150 g/day)	1772	0.11 (–0.01,0.22)	0.071	1744	0.10 (–0.02,0.23)	0.105
Nuts (25 g/day)	1769	0.05 (–1.13,1.23)	0.933	1740	0.69 (–0.38,1.75)	0.205
Cereal (50 g/day)	1774	0.02 (–0.13,0.17)	0.782	1746	–0.03 (–0.20,0.14)	0.741
Side dishes (100 g/day)	1774	0.28 (–0.00,0.55)	0.052	1746	–0.26 (–0.53,0.01)	0.058
Dairy products (200 g/day)	1774	0.13 (–0.02,0.27)	0.082	1746	0.25 (0.12,0.38)	<0.001
Cheese (30 g/day)	1768	0.22 (0.01,0.44)	0.045	1735	–0.03 (–0.28,0.21)	0.792
Eggs (60 g/day)	1763	1.09 (0.23,1.95)	0.013	1741	0.49 (–0.24,1.23)	0.188
Fat spread (5 g/day)	1763	–0.06 (–0.26,0.14)	0.558	1736	–0.03 (–0.23,0.17)	0.759
Fish (90 g/day)	1774	0.56 (–0.83,1.95)	0.428	1742	–0.08 (–1.86,1.69)	0.926
Meat and meat products	1774	0.62 (–0.03,1.26)	0.061	1746	0.65 (–0.04,1.34)	0.066
Red meat (150 g/day)	1770	0.67 (–0.62,1.96)	0.307	1740	0.80 (–0.56,2.16)	0.250
Processed meat (150 g/day)	1774	0.16 (–1.06,1.38)	0.793	1746	1.48 (0.16,2.81)	0.028
Sweets and snacks (50 g/day)	1775	0.13 (–0.07,0.33)	0.195	1746	0.31 (0.14,0.49)	0.001
Fast food (250 g/day)	1774	3.28 (1.64,4.92)	<0.001	1746	2.69 (0.75,4.63)	0.007
Low-fat milk/yoghurt	1752	0.76 (0.23,1.28)	0.005	1727	0.95 (0.42,1.47)	<0.001
Low-fat cheese/sausage	1697	0.39 (–0.10,0.89)	0.116	1698	0.65 (0.16,1.14)	0.009
Sugar in coffee/tea	1723	–0.09 (–0.59,0.40)	0.707	1677	–0.02 (–0.53,0.48)	0.930
Vegetarian	1759	–0.04 (–0.98,0.91)	0.941	1727	–0.10 (–1.12,0.93)	0.854
Energy (100 kcal/day)	1621	0.04 (–0.00,0.08)	0.059	1630	0.07 (0.03,0.11)	<0.001
Carbohydrates (10 E%)	1621	–0.09 (–0.40,0.23)	0.585	1630	–0.12 (–0.46,0.22)	0.473
Saccharose (10 g/day)	1621	–0.01 (–0.09,0.06)	0.737	1630	0.12 (0.04,0.20)	0.004
Protein (10 E%)	1621	0.44 (–0.34,1.22)	0.270	1630	0.05 (–0.76,0.85)	0.914
Fat (10 E%)	1621	0.02 (–0.37,0.42)	0.909	1630	0.18 (–0.24,0.59)	0.397
Saturated fat (10 E%)	1621	–0.04 (–0.77,0.69)	0.912	1630	0.30 (–0.45,1.06)	0.429
Fiber (10 g/day)	1621	0.19 (–0.04,0.42)	0.109	1630	0.20 (–0.05,0.44)	0.114
Healthy Eating Index	1775	0.07 (–0.21,0.34)	0.641	1746	–0.05 (-0.33,0.24)	0.757

Depicted is the regression coefficient of GWG in kg along with the 95% confidence interval. BMI: body mass index; GWG: gestational weight gain; T0: baseline assessment before the 12th week of gestation; T1: assessment after the 29th week of gestation. ^1^ linear regression models adjusted for prepregnancy BMI, age, parity, and group assignment.

## Supplementary Materials

The following are available online at https://www.mdpi.com/2077-0383/8/7/960/s1, Table S1: Detailed version of the Healthy Eating Index in the intervention and control groups.
