# Supplementary material for: Effects of a Lifestyle Intervention in Routine Care on Prenatal Dietary Behavior—Findings from the Cluster-Randomized GeliS Trial
Source: J Clin Med. 2019 Jul 2;8(7):960. doi: 10.3390/jcm8070960 (PMC6678299; doi:10.3390/jcm8070960)
Supplement: Supplementary file 1 [file jcm-08-00960-s001.pdf]

Table S1. Detailed version of the Healthy Eating Index in the intervention and control groups

	Time point	Intervention group		Control group			
		n	mean±SD	n	mean±SD	Adjusted effect size[1] (95% CI)	Adjusted p value[1]
**Score beverages**	T0	1025	95.52±12.48	979	94.29±14.86		
	T1	949	95.53±12.34	928	95.34±12.53	-0.28(-1.73,1.16)	0.703
	Time effect	p=0.834[2]		p=0.053[2]			
**Score vegetables**	T0	1022	41.68±27.69	980	39.89±28.36		
	T1	949	46.24±29.39	927	40.09±28.29	4.57(1.54,7.59)	0.003
	Time effect	p<0.001[2]		p=0.615[2]			
**Score fruit**	T0	1022	75.02±30.63	980	70.48±32.07		
	T1	948	75.42±29.94	928	71.57±31.20	2.14(1.02,3.26)	< 0.001
	Time effect	p=0.484[2]		p=0.381[2]			
**Score cereal**	T0	1025	55.62±27.60	979	53.29±26.98		
	T1	949	54.07±24.83	929	52.92±26.35	0.02(-2.3,2.4)	0.984
	Time effect	p=0.100[2]		p=0.789[2]			
**Score side dishes**	T0	1024	77.14±24.19	980	75.77±25.67		
	T1	949	77.17±24.01	929	75.26±25.39	1.29(-1.05,3.61)	0.280
	Time effect	p=0.789[2]		p=0.377[2]			
**Score nuts**	T0	1021	8.85±17.29	975	7.12±14.23		
	T1	946	10.76±19.03	925	9.39±17.18	1.05(-0.45,2.54)	0.169
	Time effect	p=0.003[2]		p<0.001[2]			
**Score dairy products**	T0	1025	52.81±36.76	979	52.67±36.45		
	T1	949	48.57±38.48	929	51.28±37.79	-2.81(-6.66,1.05)	0.153
	Time effect	p=0.002[2]		p=0.252[2]			
**Score cheese**	T0	1021	37.59±31.94	975	38.13±31.78		
	T1	944	39.70±31.07	923	38.97±31.06	0.41(-1.39,2.22)	0.653
	Time effect	p=0.064[2]		p=0.342[2]			
**Score eggs**	T0	1019	41.36±30.72	973	38.32±31.24		
	T1	945	40.32±29.18	926	38.76±29.98	-0.01(-3.5,3.4)	0.995
	Time effect	p=0.315[2]		p=0.720[2]			
**Score fish**	T0	1025	48.40±34.88	979	45.10±34.74		
	T1	947	55.34±34.58	926	46.68±33.55	7.60(5.28,9.93)	< 0.001
	Time effect	p<0.001[2]		p=0.179[2]			
**Score meat**	T0	1024	81.35±30.49	980	82.23±30.57		
	T1	949	79.85±31.81	929	80.27±30.95	0.48(-2.66,3.61)	0.767
	Time effect	p=0.319[2]		p=0.063[2]			
**Score fats**	T0	1016	99.84±3.47	977	99.76±3.10		
	T1	946	99.72±4.84	922	99.80±3.05	-0.15(-0.32,0.01)	0.074
	Time effect	p=0.538[2]		p=0.777[2]			
**Score sweets**	T0	1025	11.86±20.73	980	13.11±22.30		
	T1	949	9.58±19.52	929	8.83±19.38	0.65(-2.49,3.80)	0.684
	Time effect	p=0.002[2]		p<0.001[2]			
**Score alcohol**	T0	1023	98.28±6.81	973	97.16±9.34		
	T1	949	99.28±2.42	929	98.86±4.66	0.32(0.10,0.54)	0.004
	Time effect	p<0.001[2]		p<0.001[2]			
**Healthy Eating Index**	T0	1025	58.81±8.60	980	57.54±8.93		
	T1	949	59.33±8.21	929	57.60±8.52	1.05(-0.42,2.53)	0.162
	Time effect	p<0.001[2]		p<0.001[2]			

Abbreviations: T0: baseline assessment before the 12th week of gestation; T1: assessment after the 29th week of gestation.

[1]linear regression models fit using generalized estimating equations adjusted for pre-pregnancy BMI, age, parity and baseline score (T0);

[2]linear mixed models for repeated measures adjusted for pre-pregnancy BMI, age and parity.
